# Multilevel information storage using magnetoelastic layer stacks

**DOI:** 10.1038/s41598-019-39775-1

**Published:** 2019-02-28

**Authors:** D. P. Pattnaik, R. P. Beardsley, C. Love, S. A. Cavill, K. W. Edmonds, A. W. Rushforth

**Affiliations:** 1 School of Physics and Astronomy, University of Nottingham, Nottingham, NG7 2RD United Kingdom; 2 Department of Physics, University of York, Heslington, York YO10 5DD United Kingdom

## Abstract

The use of voltages to control magnetisation via the inverse magnetostriction effect in piezoelectric/ferromagnet heterostructures holds promise for ultra-low energy information storage technologies. Epitaxial galfenol, an alloy of iron and gallium, has been shown to be a highly suitable material for such devices because it possesses biaxial anisotropy and large magnetostriction. Here we experimentally investigate the properties of galfenol/spacer/galfenol structures in which the compositions of the galfenol layers are varied in order to produce different strengths of the magnetic anisotropy and magnetostriction constants. Based upon these layers, we propose and simulate the operation of an information storage device that can operate as an energy efficient multilevel memory cell.

## Introduction

Concepts based on magnetism hold much promise for the next generation of information storage technologies. Currently, the magnetic random access memory (MRAM) encodes information as the direction of the magnetisation of a magnetic element with lateral dimensions of order 100 nm or less^1^. The desirable characteristics of a memory device include high speed, long retention time, high storage density and energy efficient writing, storing and reading processes. Concepts based on magnetism address the first two characteristics well. The problem of achieving high storage density has been addressed by proposals to extend the device architecture to three dimensions, typically by stacking layers of magnetic materials, where each layer stores one bit of information. Examples include multibit memory cells^2^, the magnetic soliton ratchet^3^, the Extra-ordinary Hall effect balance^4^ and the 3-dimensional magnetic abacus^5^. These proposed concepts use either magnetic fields or electrical currents to manipulate the magnetisation and thereby write the information. This is costly in terms of energy, generates heat and limits the ability to address individual bits without cross-talk to neighbouring bits. It has been recognised that using electric fields to manipulate magnetisation would provide a more energy efficient alternative method^6,7^. One route is to couple the electric field to the magnetisation in a hybrid piezoelectric/magnetic structure, whereby the electric field induces a mechanical strain that is transmitted to the magnetic element, modifying the magnetic anisotropy through inverse magnetostriction, which causes the orientation of the magnetisation to switch^6–11^. Recently, we have employed this technique to demonstrate voltage-induced non-volatile switching of the magnetisation in a device incorporating an epitaxial layer of galfenol (Fe_81_Ga_19_)^12^. Epitaxial galfenol possesses biaxial anisotropy and a large magnetostriction coefficient which make it suitable for switching the magnetisation between the orthogonal stable easy axis directions. Here we extend this concept to a hybrid piezoelectric/ferromagnetic multilayer device consisting of two epitaxial galfenol layers separated by a non-magnetic metallic spacer. The galfenol layers are designed to possess different magnitudes of magnetocrystalline anisotropy and of the magnetostriction coefficient, so that the magnetisation switching will occur at different values of voltage induced strain for each layer. We demonstrate experimentally how the application of a voltage to the piezoelectric layer generates a mechanical strain that modifies the magnetic anisotropy of the two galfenol layers by different magnitudes. We also discuss the design and operation of a multilevel memory device based upon galfenol layers.

**Figure 1:**
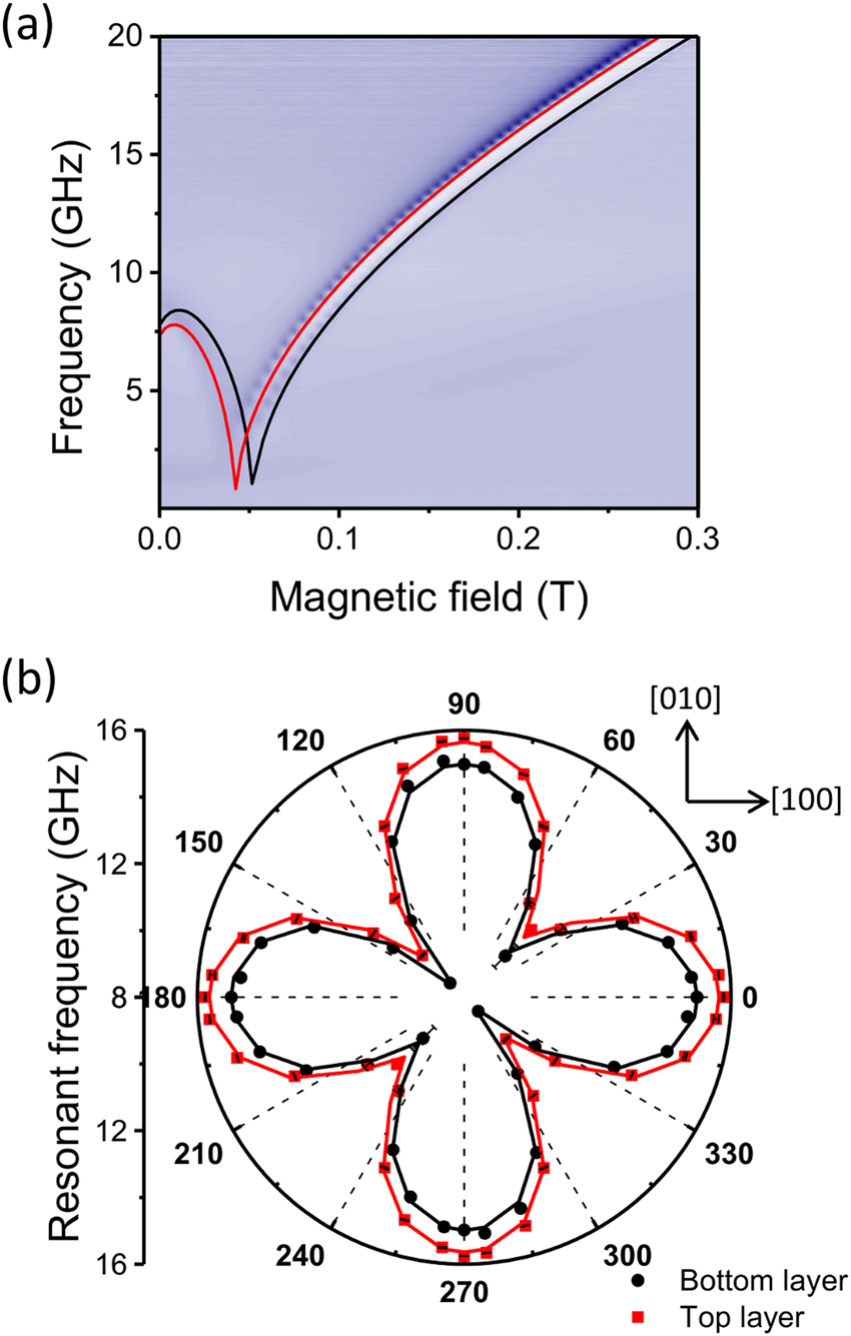
Ferromagnetic resonance. (**a**) The microwave transmission as a function of frequency and magnetic field applied along the [1 $\bar{1}\,$0] direction. (**b**) Resonant frequency as a function of magnetic field angle measured at a fixed field magnitude of 0.1 T. The angle is with respect to the [100] direction. Points represent the experimental data. In both (**a**,**b**) black and red curves represent the fit to the data.

The magnetic multilayer stack consists of layers in the sequence Fe_84_Ga_16_[5 nm]\Cu[5 nm]\Fe_88_Ga_12_[5 nm]\Al[5 nm] deposited onto a 150 μm thick GaAs substrate using magnetron sputtering. The magnetic properties of the layers were characterised using ferromagnetic resonance (FMR) measurements. An unpatterned 5 mm × 5 mm sample was mounted face down onto a coplanar waveguide (CPW) with an external magnetic field applied in the plane of the layers. Figure 1(a) shows a 2D FMR map of the microwave transmission, as a function of frequency and applied magnetic field. The presence of two distinct resonances indicates two layers with different magnetic parameters. Figure 1(b) shows the resonant frequency as a function of magnetic field angle measured at a fixed field magnitude of 0.1 T. The dependence on angle shows that both layers possess cubic magnetocrystalline anisotropy favouring the [100]/[010] axes, plus a small uniaxial anisotropy component favouring the [110] direction, typical of magnetic layers deposited onto GaAs^13,14^.

An expression for the FMR frequency is given by Smit and Beljers^15^, and shown in Eq. (1),

1 $${(\frac{\omega }{\gamma })}^{2}=\frac{1}{{M}_{s}^{2}si{n}^{2}\theta }[\frac{{{\rm{\partial }}}^{2}F}{{\rm{\partial }}{\theta }^{2}}\frac{{{\rm{\partial }}}^{2}F}{{\rm{\partial }}{\phi }^{2}}-{(\frac{{{\rm{\partial }}}^{2}F}{{\rm{\partial }}\theta {\rm{\partial }}\phi })}^{2}],$$

where *M*_*s*_ is the saturation magnetisation, ω the resonant angular frequency, γ the gyromagnetic ratio and $\theta$ and $\phi$ are the polar and in-plane azimuthal angles of the magnetisation, respectively, which can be calculated with the equilibrium conditions,

2 $$\frac{\partial F}{\partial \theta }=0,\frac{\partial F}{\partial \phi }=0,$$

3 $$\begin{array}{c}F=H{M}_{s}(sin\theta \,\cos ({\phi }_{H}-\phi ))+{M}_{s}^{2}co{s}^{2}\theta +\frac{1}{8}{M}_{s}{H}_{C}(si{n}^{4}\theta si{n}^{2}2\phi +si{n}^{2}2\theta )\\ \,\,\,+\frac{1}{2}{M}_{s}{H}_{U}si{n}^{2}\theta co{s}^{2}(\phi -\frac{\pi }{4})\end{array}$$

The magnetic free energy density, F, is an expression consisting of the cubic (*H*_*C*_) and uniaxial (*H*_*U*_) magnetic anisotropy fields, the external magnetic field (*H)*, the magnetisation (*Ms)*, and the demagnetising field, favouring magnetisation lying in the plane of the film. By solving Eq. (1) under the conditions of Eq. (2), theoretical curves for the resonant frequency as a function of applied field were obtained for each layer and are in excellent agreement with the experimental data in Fig. 1(a,b). The fitting yields the anisotropy constants, *K*_*C*_ = *μ*_0_*MH*_*C*_ = 33.0 ± 0.3 kJm^−3^ (32 ± 0.3 kJm^−3^) and *K*_*U*_ = *µ*_0_*MH*_*U*_ = 4.1 ± 0.5 kJm^−3^ (6.2 ± 0.5 kJm^−3^), and saturation magnetisation, *μ*_0_*M*_*s*_ = 1.87 ± 0.01 T (1.78 ± 0.01 T) for the top (bottom) layer of the stack. The assignment of the layers is based on the fact that the top layer is designed to have the larger concentration of iron and therefore, has the larger saturation magnetisation. The effect of the larger *M*_*s*_ on the resonant frequency is dominant compared to the small changes in anisotropy leading to unambiguous assignment of the layers.

**Figure 2:**
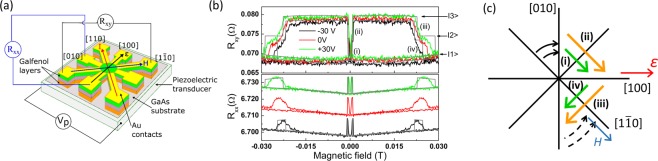
Magnetotransport investigations. (**a**) Schematic of the measurement setup. A voltage V_P_ applied to the transducer induces a strain ε along the [100] direction. (**b**) Transverse (R_xy_) and longitudinal (R_xx_) resistances measured as a function of magnetic field, H, applied along the [1 $\bar{1}\,$0] direction for V_P_ = −30 V, 0 V and +30 V. Gray lines represent modelled data for V_P_ = −30 V and +30 V. Transitions labelled (i–iv) represent abrupt switching of the magnetisation as shown schematically in (**c**), where green/orange arrows represent switching of the magnetisation directions in the bottom/top galfenol layers respectively, and black arrows represent coherent rotations of the magnetisation. The magnetisation switching sequence gives rise to 3 distinct levels of the transverse resistance, labelled |1>, |2> and |3>.

Magnetotransport measurements of the longitudinal (R_xx_) and transverse (R_xy_) resistances were used to provide electrical readout of the magnetisation states of the layers as a function of strain and external magnetic field. A magnetotransport device (Fig. 2(a)) was fabricated in the geometry of a cross and was bonded to a piezoelectric transducer capable of inducing a uniaxial strain of order 10^−4^ for applied voltages in the range −30 V to +30 V. Uniaxial strain was induced along the [100] crystal direction with tensile strain defined as positive along this direction. The external magnetic field was applied in the plane of the device parallel to the [1 $\bar{1}\,$0] direction. The magnetotransport measurements were carried out with the voltage applied to the piezoelectric transducer simultaneously.

Figure 2(b) shows the measured longitudinal and transverse resistances respectively. Abrupt changes in the resistance values occur when the magnetisations of individual layers switch direction. There are two magnetisation dependent contributions to the resistance. The anisotropic magnetoresistance (AMR) for a single magnetic layer depends on the angle β between the magnetisation and the current, as described by Eq. (4), while the transverse component of the AMR, also known as the planar Hall effect, is described by Eq. (5), where *ΔR* is the amplitude of the AMR and *R*_0_ is the magnetisation independent contribution to the longitudinal resistance.

4 $${R}_{xx}={R}_{0}+{\rm{\Delta }}Rcos2\beta $$

5 $${R}_{xy}={\rm{\Delta }}Rsin2\beta $$

The longitudinal resistance will also contain a contribution, ${R}_{xx}^{GMR}$, arising from the giant magnetoresistance (GMR) which depends upon the relative angle *δ* between magnetisations of the two galfenol layers, as given by Eq. (6)^16^:

6 $${R}_{xx}^{GMR}={\rm{\Delta }}{R}^{GMR}\cdot \frac{(1-cos\delta )}{2}$$

The 5 nm Cu spacer layer allows for a measurable contribution to the GMR, of amplitude ${\rm{\Delta }}{R}^{GMR}$, whilst preventing significant exchange coupling between the layers^17^.

Minimising *F* in Eq. (3) using the values of *K*_*C*_ and *K*_*U*_ extracted from fitting to the FMR data yields the angles of the magnetisations (φ = *β* +  $\frac{\pi }{4}$) as a function of the applied magnetic field. Modelling the magnetoresistance of the two layers as two resistors in parallel produced simulated resistance curves that resemble the main features observed in the measured resistances. This process required the introduction of a uniaxial magnetic anisotropy energy to Eq. (3) for each layer, favouring the [100] axis to represent the effect of the strain. Determination of the magnitude of this contribution will be discussed below. The simulated curves are shown by the grey lines in Fig. 2(b), and the switching sequences for the two layers are illustrated schematically in Fig. 2(c). Sweeping the magnetic field along the [1 $\bar{1}\,$0] axis from high negative values, the magnetisations of both layers rotate coherently towards the [010] direction. At magnetic fields close to zero, the magnetisations of the layers switch abruptly towards the [100] direction, which is favoured by a uniaxial magnetic anisotropy induced by the tensile strain along [100]. Transitions labelled (i) and (ii) correspond to this switch, which occurs at a slightly different value of the magnetic field in the two galfenol layers due to their different magnetic properties. The contributions of the AMR and GMR to the magnetotransport data can be separately and unambiguously identified due to their different phenomenology. The change of the transverse resistance arises from the AMR, whereas the change in the longitudinal resistance arises mostly due to the GMR, manifesting as an increase in the resistance when the magnetisation of the first galfenol layer switches direction (transition (i)), resulting in an increase in δ. The resistance decreases when the magnetisation of the second galfenol layer changes direction (transition (ii)), resulting in a decrease in δ. As the magnitude of the magnetic field increases further the magnetisations of both layers rotate slightly away from the [100] direction until, at approximately 17 mT, they switch abruptly towards the [0 $\bar{1}\,$0] axis. Transitions labelled (iii) and (iv) represent this transition for the two galfenol layers, with the resultant features arising due to the AMR and GMR in the transverse and longitudinal resistances respectively. The transitions in the experimental data are not as abrupt as those produced in the simulated curves. This is likely due to the fact that the simulations model a magnetic domain with uniform magnetic properties, whereas in the experimental device it is likely that the magnetisation switching occurs by the nucleation and propagation of domain walls which will occur over a range of applied magnetic fields for different parts of the device.

The effect of varying the uniaxial strain is to move transitions (i) and (ii) to lower fields and transitions (iii) and (iv) to higher field values as the tensile strain is increased (voltage applied to the transducer is made more positive). This is consistent with the understanding that the tensile strain makes the [100] axis the easier axis. The fact that the switching fields for the two galfenol layers have different dependences on the voltage induced strain indicates that the layers have different magnetoelastic constants due to their different compositions. An additional contribution may arise from the different positions of the layers within the stack. The upper layer is further away from the piezoelectric transducer, so the strain transmitted to that layer may be reduced compared with that transmitted to the lower layer. The magnetoelastic coefficient, *B*_1_, connects the uniaxial strain with the induced change in the magnetic free energy $({\rm{\Delta }}F)$, as shown by Eq. (7):

7 $${\rm{\Delta }}F={B}_{1}({\varepsilon }_{xx}-{\varepsilon }_{yy})si{n}^{2}\alpha $$

where ${B}_{1}=\frac{3}{2}{\lambda }_{100}({c}_{12}-{c}_{11})\,$is the magnetoelastic constant, ${\lambda }_{100}$ is the magnetostriction constant, *c*_12_ and *c*_11_ are the elastic constants, *ε*_*xx*_ and *ε*_*yy*_ are the relevant components of the strain tensor, and *α* is the angle between the magnetisation and the strain.

Equation (7) is an additional contribution to the magnetic free energy in Eq. (3). We are unable to fit the model to our data in order to extract the values of *B*_1_ because we do not have an independent measurement of the uniaxial strain for our device. However, using reasonable estimates of *B*_1_=1 × 10^7^ Jm^−3^ for the bottom galfenol layer, which is close to the 19% Ga composition^18^ and *B*_1_ = 0.26 × 10^7^ Jm^−3^ for the top layer with reduced Ga composition, and for values of *ε*_*xx*_ − *ε*_*yy*_ = 0.54 × 10^−4^ and 0.83 × 10^−4^ representing the induced strain for voltages −30 V and +30 V respectively^7^, we are able to simulate switching fields in good agreement with the experiment.

### Application as a memory device

**Figure 3:**
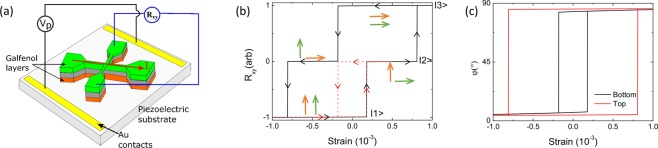
A memory cell based on magnetoelastic multilayers. (**a**) Schematic of the proposed device consisting of a cross structure on a piezoelectric layer. Voltage applied to the contacts on the piezoelectric layer induce switching of the magnetisation between orthogonal directions in the magnetic layers via the magnetoelastic coupling. Readout is via the transverse resistance which has n + 1 distinct levels for a n-layer device. (**b**) The modelled transverse resistances of a device based on the magnetic bilayer structure investigated in our study. The red dashed curve represents a minor loop to show that state |2> is non-volatile. Green/orange arrows represent the directions of the magnetisation in the bottom/top galfenol layer respectively for each resistance state. (**c**) The angles of the magnetisations of the top and bottom layers as a function of the induced strain corresponding to the black curve in (b).

Using the parameters discussed above, we have modelled the characteristics of a memory device (see schematic device design in Fig. 3(a)) for which the magnetisation of each galfenol layer can be switched between the [100] and [010] directions by strain alone. Figure 3(b) shows the calculated transverse resistance as a function of the induced strain, based upon the calculated dependence of the angle of the magnetisation of each layer, shown in Fig. 3(c). The magnetic state can be set such that the transverse resistance switches between three non-volatile values, which can represent three distinct states (|1>, |2> and |3>) of a memory device. These are the same three states identified in the experimental measurement of the transverse resistance, shown in Fig. 2(b). This concept for information storage, which can be extended to multiple magnetic layers, is similar to the magnetic abacus proposed by Zhang *et al*.^5^, except that here we use the planar Hall effect for read out and the write operation is carried out using voltage-induced strain instead of magnetic field or current induced torque. In our experimental device, the use of a mm-sized commercial piezoelectric transducer is convenient as it is compatible with the studied galfenol/GaAs layers, but does not allow us to achieve the range of strain necessary to demonstrate this strain-induced switching. However, the size of the device can be reduced to the nanoscale by depositing the magnetic stack directly onto a piezoelectric substrate and applying the voltage via gate electrodes patterned on the surface, as depicted in Fig. 3(a). The use of surface electrodes has been demonstrated to induce strain of order 10^−3 ^^19^ with the application of a few tens of Volts to electrodes patterned on the µm scale. It has been calculated^20^ that with surface electrodes patterned on the sub-µm scale, the same strain could be generated with the application of less than 1V and the energy cost of applying the voltage pulses would be on the order of a few aJ. This should be compared with the fJ operation of modern MRAM operated using spin transfer torque, thus demonstrating the potential for hybrid piezoelectric/magnetic stacks to enable the design of high density, ultra-energy efficient information storage devices.

In conclusion, we have investigated the magnetic reversal characteristics of a galfenol/spacer/galfenol layer stack where the top and bottom galfenol layers have different compositions. We found that the galfenol layers have different magnitudes of magnetocrystalline anisotropy and different values for the magnetoelastic coefficient. This leads to distinct magnetic reversal switching fields for each layer, which can be tuned by an applied uniaxial stress. Magnetotransport measurements allow the switching of the magnetisation in each layer to be detected independently. We propose and simulate the operating characteristics of an energy efficient multilevel memory cell based on a magnetoelastic layer stack.

## Methods

The Fe_84_Ga_16_[5 nm]\Cu[5 nm]\Fe_88_Ga_12_[5 nm]\Al[5 nm] stack was deposited onto a 150 μm thick GaAs substrate using magnetron sputtering. The substrate was first prepared by etching in dilute hydrochloric acid and annealed in vacuum at 500 °C for 1 hour before cooling to room temperature for the deposition of the layers. Devices for magnetotransport measurements were fabricated in the geometry of a cross structure using photolithography and argon ion milling. The magnetotransport device was bonded to a piezoelectric transducer capable of inducing a uniaxial strain of order a few 10^−4^ for applied voltages in the range −30 V to +30 V. Uniaxial strain was induced along the [100] crystal direction with tensile strain defined as positive along this direction. For ferromagnetic resonance (FMR) measurements, an unpatterned 5 mm × 5 mm sample was mounted face down onto a 50 Ω coplanar waveguide (CPW) connected to a two port Rhode and Schwarz vector network analyser (VNA) and centred between the poles of an electromagnet. By measuring the microwave transmission losses whilst sweeping the microwave frequency as a function of bias magnetic field and azimuthal angle, angular dependent FMR spectra were collected.
